# [^18^F]F13640: a selective agonist PET radiopharmaceutical for imaging functional 5-HT_1A_ receptors in humans

**DOI:** 10.1007/s00259-022-06103-1

**Published:** 2023-01-19

**Authors:** Pierre Courault, Sophie Lancelot, Nicolas Costes, Matthieu Colom, Didier Le Bars, Jérôme Redoute, Florent Gobert, Frédéric Dailler, Sibel Isal, Thibaut Iecker, Adrian Newman-Tancredi, Inés Merida, Luc Zimmer

**Affiliations:** 1 Université Claude Bernard Lyon 1, CNRS, INSERM, Lyon Neuroscience Research Center, Lyon, France; 2 Hospices Civils de Lyon (HCL), Lyon, France; 3 CERMEP, Bron, France; 4 Neurolixis, Castres, France

**Keywords:** [^18^F]F13640, 5-HT_1A_ receptors, Functional receptor, PET imaging, Brain, Modeling study

## Abstract

**Purpose:**

F13640 (a.k.a. befiradol, NLX-112) is a highly selective 5-HT_1A_ receptor ligand that was selected as a PET radiopharmaceutical-candidate based on animal studies. Due to its high efficacy agonist properties, [^18^F]F13640 binds preferentially to functional 5-HT_1A_ receptors, which are coupled to intracellular G-proteins. Here, we characterize brain labeling of 5-HT_1A_ receptors by [^18^F]F13640 in humans and describe a simplified model for its quantification.

**Methods:**

PET/CT and PET-MRI scans were conducted in a total of 13 healthy male volunteers (29 ± 9 years old), with arterial input functions (AIF) (*n* = 9) and test–retest protocol (*n* = 8). Several kinetic models were compared (one tissue compartment model, two-tissue compartment model, and Logan); two models with reference region were also evaluated: simplified reference tissue model (SRTM) and the logan reference model (LREF).

**Results:**

[^18^F]F13640 showed high uptake values in raphe nuclei and cortical regions. SRTM and LREF models showed a very high correlation with kinetic models using AIF. As concerns test–retest parameters and the prolonged binding kinetics of [^18^F]F13640, better reproducibility, and reliability were found with the LREF method. Cerebellum white matter and frontal lobe white matter stand out as suitable reference regions.

**Conclusion:**

The favorable brain labeling and kinetic profile of [^18^F]F13640, its high receptor specificity and its high efficacy agonist properties open new perspectives for studying functionally active 5-HT_1A_ receptors, unlike previous radiopharmaceuticals that act as antagonists. [^18^F]F13640’s kinetic properties allow injection outside of the PET scanner with delayed acquisitions, facilitating the design of innovative longitudinal protocols in neurology and psychiatry.

**Trial Registration.:**

Trial Registration EudraCT 2017–002,722-21.

**Supplementary Information:**

The online version contains supplementary material available at 10.1007/s00259-022-06103-1.

## Introduction

Serotonin (5-hydroxytryptamine, 5-HT) is known to have a variety of functions in the central nervous system, which are mediated by a diversity of receptors. Among them, the serotonin 1A receptor subtype (5-HT_1A_) is a G-coupled protein receptor (GCPR) which has attracted extensive interest because it is involved in regulation of mood, cognition, pain, and movement [1]. Consequently, various positron emission tomography (PET) radiotracers have been developed to target 5-HT_1A_ receptors, including [O-*methyl*-^11^C]WAY100635 [2], [*carbonyl*-^11^C]WAY100635 [2, 3], and [^18^F]MPPF, a fluorinated derivate of WAY100635 [4, 5]. [^18^F]MPPF binding is observed mainly in brain regions with a high density of 5-HT_1A_ receptors such as hippocampus and raphe nuclei [5–7] and it has been used to explore various psychiatric and neurologic diseases such as epilepsy [8], narcolepsy [9], Alzheimer’s disease [10], or multiple system atrophy [11]. Other derivatives of WAY100635 have been also proposed as radiopharmaceuticals but have been less used in human [12].

Nevertheless, although [^11^C]WAY100635 and [^18^F]MPPF are widely used for in vivo exploration of 5-HT_1A_ receptors, PET imaging using these radiopharmaceuticals remains limited in terms of pathophysiological interpretation because of their antagonist pharmacological properties. Indeed, antagonists bind both G protein-coupled and G-protein uncoupled receptors with the same affinity, labeling the total receptor population, regardless of its functional status. In contrast, agonists have higher affinity for their target GPCRs when the latter are coupled to G-proteins, i.e., they are in a functional state which is directly associated with neurotransmission [13, 14]. Thus, we hypothesized that highly specific 5-HT_1A_ receptor agonist radiotracers would constitute useful tools to explore endogenous serotonergic neurotransmission and pathophysiological changes that specifically affect functional receptors and which would not be detectable using antagonist radiopharmaceuticals [15].

Very few 5-HT_1A_ agonists have been used as chemical templates to develop a PET radiopharmaceutical. [^11^C]CUMI-101, which was initially presented as an agonist, was later found to act as a partial agonist or even as an antagonist [16], and it also binds to α_1_ adrenoceptors [17]. These suboptimal pharmacological properties explain its modest sensitivity to pharmacological challenges or to endogenous serotoninergic changes in human [18, 19]. In this context, we chose to use highly specific and pharmacologically well-characterized 5-HT_1A_ receptor agonists to develop the corresponding radiopharmaceuticals. Following several studies, we selected F13640 (a.k.a. befiradol or NLX-112), which possesses high affinity (nanomolar Ki) for 5-HT_1A_ receptors, high selectivity (> 1000-fold) over a large range of other CNS targets and whose chemical structure includes a fluorine which is substitutable by a fluorine-18. As a result, [^18^F]F13640 was proposed as the first preclinical fluorinated 5-HT_1A_ receptor agonist radiopharmaceutical, supported by compelling preclinical data in animal models [20, 21]. Although a first image obtained in a healthy volunteer suggested favorable brain penetration by [^18^F]F13640 [22], it remained to be demonstrated whether [^18^F]F13640 could become a usable radiopharmaceutical for future clinical investigation. The objectives of the present study were, therefore, to perform a full PET kinetic modeling of [^18^F]F13640 using arterial input function (AIF), to identify a reference region suitable for a simplified modeling method, and to assess reproducibility with test–retest scans. Ultimately, the present data characterizing [^18^F]F13640 as a selective agonist radiopharmaceutical will, for the first time, enable the investigation of changes in both 5-HT_1A_ receptor expression and functionality in patients suffering from disorders arising from dysfunctional serotonergic neurotransmission.

## Material and methods

### Synthesis and quality control

[^18^F]F13640 (3-chloro-4-fluorophenyl)-[4-^18^fluoro-4-[[(5-methylpyridin-2-yl)methylamino]methyl]piperidin-1-yl]methanone; a.k.a. [^18^F]NLX-112 or [^18^F]befiradol) was synthesized as previously described [21]. Briefly, radiolabeling was obtained by a nucleophilic fluoro-substitution on the F13640 nitro precursor using an automated radiosynthesizer (Neptis, ORA). Chemical and radiochemical purity measured by HPLC was higher than 95%. Mean molar activity (EOS) was 82 ± 18 GBq/µmol.

### Study design

The study was performed according to the ethical standards of the institutional and national research committee and with the principles of the 1964 Declaration of Helsinki. The study was approved by a French ethics committee (Eudra-CT: 2017–002,722-21) and pre-registered on the ClinicalTrials.gov database (NCT03347331).

**Fig. 1:**
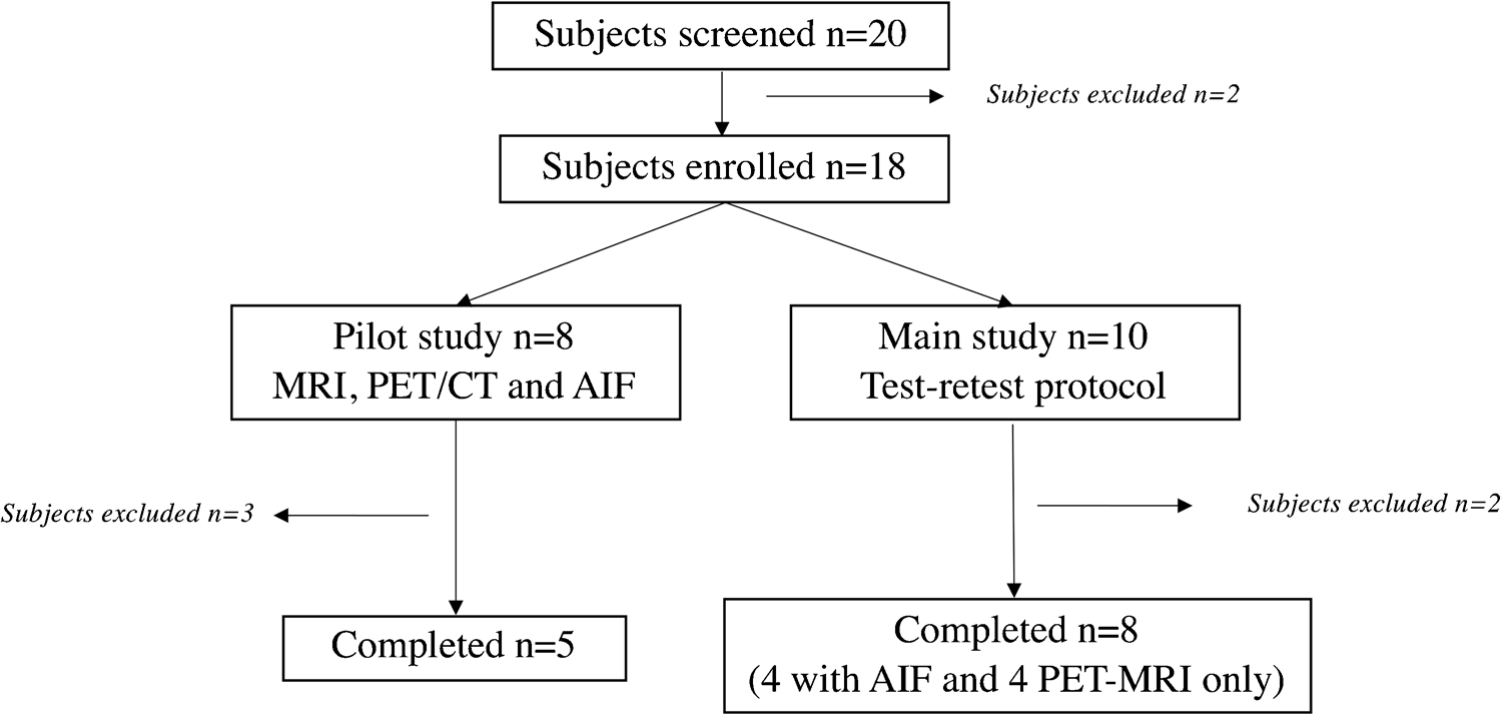
Summary flow-chart of healthy subjects’ inclusion for the pilot study and main test–retest study. Analysis focused only on the 8 PET-MRI subjects and 4 PET-MRI subjects when AIF is considering

Participants were healthy volunteers, i.e., without neurological or psychiatric disorders, active infectious disease, severe and progressive medical pathology, without addiction (smoking, cannabis, alcohol…), or MRI and PET contraindications. Twenty volunteers were screened in the study and signed consent. Two volunteers failed inclusion due to exclusion criteria (MRI contraindication and body weight superior to 90 kg). The first eight volunteers were included in a pilot study in which the subjects underwent an anatomical MRI scan (3D T1-weighted sequence on a 1.5-T Siemens Magneton scanner, Siemens AG, Erlanger, Germany) and a 90 min PET/CT (Siemens Biograph mCT64) scan with arterial input function (AIF, data not shown). The later 10 volunteers were included to perform a test–retest protocol with two PET-MRI scans as described below. Four participants had AIF measurements on one of the two visits. Two volunteers were lost to follow-up. To sum up, data analysis focused on four participants when concerning PET modeling with AIF and eight participants for test–retest analysis. Figure 1 summarizes recruitment of healthy subjects in the study.

### PET-MRI test–retest protocol

Participants underwent a PET-MRI acquisition on the Siemens mMR Biograph system. Because the pilot study group scans showed that [^18^F]F13640 activity curves slowly continued to vary even at later time points, suggesting that there may be further changes beyond 90 min. A PET-MRI acquisition of almost 4 h was tested to observe kinetics for a longer period. For the comfort of the subjects, the acquisition was carried out in two parts. In part 1 (PET1), list-mode PET data were acquired for 90 min directly after the injection of [^18^F]F13640 (150 MBq + 1 MBq/kg ± 10%) (PET1, [0; 90] min. post injection). Subjects were then taken out of the camera for a break. One hour later, for part 2 (PET2), subjects had a second PET-MRI scan for 75 min (PET2; [150; 225] min. post injection). Participants performed this PET-MRI protocol twice (test and retest sessions) 1 to 9 weeks apart.

During PET1, a 3D T1 MPRAGE MRI was acquired in sagittal orientation, with a matrix size of 256 × 256 × 176 and a voxel size of 1 mm iso. TR/TE was 3300/2.45 ms, TI 1100 ms, and flip angle 8°. A quicker T1 MPRAGE MRI was acquired at the beginning of PET2 to accurately register data from the two sessions (sagittal acquisition, matrix size 256 × 256 × 176, voxel size 1 mm iso, TR/TE 1800/2.34 ms, TI = 850 ms, flip angle 8).

### PET-MRI image processing

The PET data from the two parts were reconstructed independently. First, a MR-based attenuation correction map was generated from the T1 MPRAGE of each part [23]. PET1 and PET2 list modes were then corrected for motion with the EBER algorithm [24]. PET1 was rebinned into 24 frames of variable duration (8 × 15 s, 3 × 60 s, 5 × 120 s, 1 × 300 s, 7 × 600 s), and PET2 was rebinned into 8 frames (7 × 600 s, 1 × 300 s). Sinograms were normalized and corrected for attenuation [23], scatters, and randoms. PET reconstructions were performed with Siemens e7tools using the OP-OSEM algorithm with PSF, 3 iterations, and 21 subsets. A matrix size of 256 and a zoom of 3 were applied yielding a voxel size of 0.93 × 0.93 × 2.03 mm with a 4 mm 3D post-reconstruction gaussian filtering.

Both dynamic PET series from PET1 and PET2 were combined in a single dynamic series in the following way. The mean of each dynamic PET series was computed. The mean of PET2 was rigidly coregistered to the mean of PET1. This rigid transformation was applied to all frames of PET2 to obtain the PET2 frames aligned with the PET1 frames. Decay correction was applied to PET2 and PET1 setting the reference time for both series to the start time of the PET1 scan. PET1 and PET2 were finally concatenated in a single and harmonized dynamic 4D file. These preprocessing steps were performed with the minc toolkit functions (http://bic-mni.github.io).

In addition to the above, the T1 image of the retest session and the T1 image of the test session were coregistered by applying the computed rigid coregistration matrix to all images of the retest session. The structural T1 image from the test session was automatically segmented into anatomical regions using the multi-atlas propagation with enhanced registration (MAPER) method [25] and the 95-region Hammersmith atlas [26–28]. The raphe nucleus was segmented based on functional data from a previous study [29]. The regional segmentation was projected to test and retest sessions, and regional time activity curves (TAC) were extracted for a selection of brain regions. Regions selected for analysis were cortical regions (cingulate, frontal, occipital, parietal, temporal superior, and temporal inferior), amygdala, central grey nuclei, hippocampus, insula, parahippocampal gyrus, thalamus, brainstem, dorsal and median raphe nuclei, cerebellum (total, grey matter, and white matter), vermis, frontal lobe white matter, and corpus callosum.

### Arterial input function (AIF)

Four participants underwent AIF measures during their test or retest session. AIF, free fraction, and metabolites were measured based on 29 blood samples. Samples were collected manually after arterial catheterization with local anesthesia (lidocaine patch 5%). Sample time points were as follows: every 5 s in the first minute, every 15 s in the second minute, every 30 s in the third minute, and at time 5, 10, 20, 30, 40, 60, 75, 90, 160, and 205 min. Whole blood radioactivity was counted on every sample using a gamma counter (Perkin-Elmer) to measure the whole blood curve (Cwb). Plasma was collected and counted after centrifugation (4 min, 3000 G at 4 °C) on 18 out of 29 samples (*t* = 15 s and every sample after the first minute to calculate plasma to whole blood ratio *f*_*wb*_). Uncorrected plasma curve (*Cp*) was determined by the mean *f*_*wb*_ and the whole blood curve: *Cp* (*t*) = *f*_*wb*_*.Cwb* (*t*). On 5 samples (*t* = 2, 10, 30, 90, and 205 min) free fraction and metabolites were determined. For metabolites, 500 µL of plasma was added to 750 µL of acetonitrile with cold carrier of F13640 at 20 mg/L, centrifuged (4 min, 3000 G at 4 °C), filtered at 0.45 µm, diluted with water, and injected in a C8 HPLC column with a mixed mobile phase water/acetonitrile/TFA (60/40/0.1). Metabolites and [^18^F]F13640 were separated, and fractions were collected and counted in the gamma counter. The activity of the [^18^F]F13640 fraction was divided by the total activity recovered from the gamma counter to give the plasma parent fraction of unmetabolized [^18^F]F13640 (PPf). For plasma free fraction (*fp*), 1 mL of plasma was centrifugated (Centrifree®, Millipore) for 20 min, 2000 g at 20–25 °C, and 100 µL of ultrafiltrate plasma was counted in a gamma counter. After counting, all samples were weighed, and counts were corrected. The *fp* was calculated from the ratio of concentrations in the ultrafiltrate and whole plasma. The AIF was derived from *Cp* (*t*) according to: *AIF* (*t*) = *PPf* (*t*)*.Cp* (*t*)*.fp* (*t*)*.*

### Kinetic modeling

Cerebral TACs (*PET* (*t*)) were modeled with three different AIF models for participants who performed AIF (*n* = 4): one-tissue compartment (1TC), two-tissue compartment (2TC), and the Logan graphical method (LOGAN) [30]. Blood volume in tissue (*V*_*b*_) was included as a model parameter in the operational equation according to: *PET* (*t*) = *V*_*b*_*.Cwb* (*t*)* x* + (*1-V*_*b*_).*C*_*T*_ (*t*), where *C*_*T*_ is the TAC in tissue*.* Model fit accuracies were compared using the Akaike Information Criterion (AIC). Two models with reference region were also assessed: the Simplified Reference Tissue Model (SRTM) [31] and the logan reference model (LREF) [30]. Four different reference regions were compared: corpus callosum (CC), cerebellum (CER), white matter cerebellum (CERWM), and frontal lobe white matter (FLWM). Distribution volume ratio (DVR) for models with AIF (DVR_1TC_ or DVR_2TC_) was calculated as the ratio between the Vt of the region of interest and the Vt of the reference region. DVRs for SRTM (DVR_SRTM_) were calculated by adding one to the binding potential (BP) value. DVRs calculated with AIF models were compared to DVRs obtained with reference region models using linear regression to determine fitting parameters: intercept, slope, and determination coefficient (*R*^2^). Kinetic modeling was done using the Turku PET center utilities library (TPCCLIB, https://gitlab.utu.fi/vesoik/tpcclib).

### Test–retest reproducibility and reliability

For all the participants in the test–retest study (*n* = 8), models with reference region were applied on both sessions. Bias and variability (VAR) were calculated to assess reproducibility, and the intraclass correlation coefficient (ICC) was computed to estimate reliability. Bias was calculated as (DVR_retest_ − DVR_test_)/DVR_test_ × 100 and VAR as the standard deviation (SD) of the bias. Parameters were expressed as percentage. ICC was calculated as (BSMSS-WSMSS)/(BSMSS + WSMSS) where the BSMSS is the mean sum of the square between subjects and WSMSS is the mean sum of the square within subjects [32].

### Statistical analysis

Statistical analysis was performed using RStudio (RStudio Team 2020, http://www.rstudio.com/). Paired Student’s *t*-tests were used to assess differences between injected doses and molar activities between test and retest sessions. Significant threshold was set at *p* < 0.05.

## Results

### Subject demographics

Mean age of the participants was 29 ± 9 years in the test–retest study. No significant differences were found in the activity of the [^18^F]F13640 doses administered to the subjects or in the molar activities of [^18^F]F13640 between the test and retest (*p* value > 0.05). Due to an injection error, participant 8 received a dose of [^18^F]F13640 for a retest session that was slightly lower than the recommended dosages described in the study. Details are presented in Table 1.

**Table 1:** Age, injected dose, and molar activity per subject and test and retest session. No significant differences were found between test and retest sessions for injected dose and molar activity (both *p* value > 0.05)

	Test session			Retest session	
Subject	Age	Activity injected (MBq)	Molar activity (GBq/µmol)	Activity injected (MBq)	Molar activity (GBq/µmol)
1	25	211	75	214	79
2	28	213	71	219	83
3	23	213	75	224	108
4	23	210	78	227	76
5	45	232	72	257	126
6	21	210	87	222	81
7	42	226	75	243	105
8	27	243	124	188	76
Mean ± SD	29 ± 9	220 ± 12	82 ± 18	224 ± 20	92 ± 19

### Modeling study

Mean plasma parent fractions were 99.40% ± 0.00%, 97.40% ± 0.01%, 96.20% ± 0.01%, 95.40% ± 0.01%, and 95.30% ± 0.01% at 2, 10, 30, 90, and 205 min respectively after injection. One value was discarded for participant 2 at 90 min due to non-interpretable value. Mean plasma parent fraction was modeled with a one-exponential function:

$$\mathrm{PP}f\left(t\right)={1-A}_{0}.(1-{e}^{\left(-\mathrm{ln}\left(2\right)*\frac{t}{T}\right)})$$

with *A*_0_ = 0.046 and *T* = 9.06 min

**Fig. 2:**
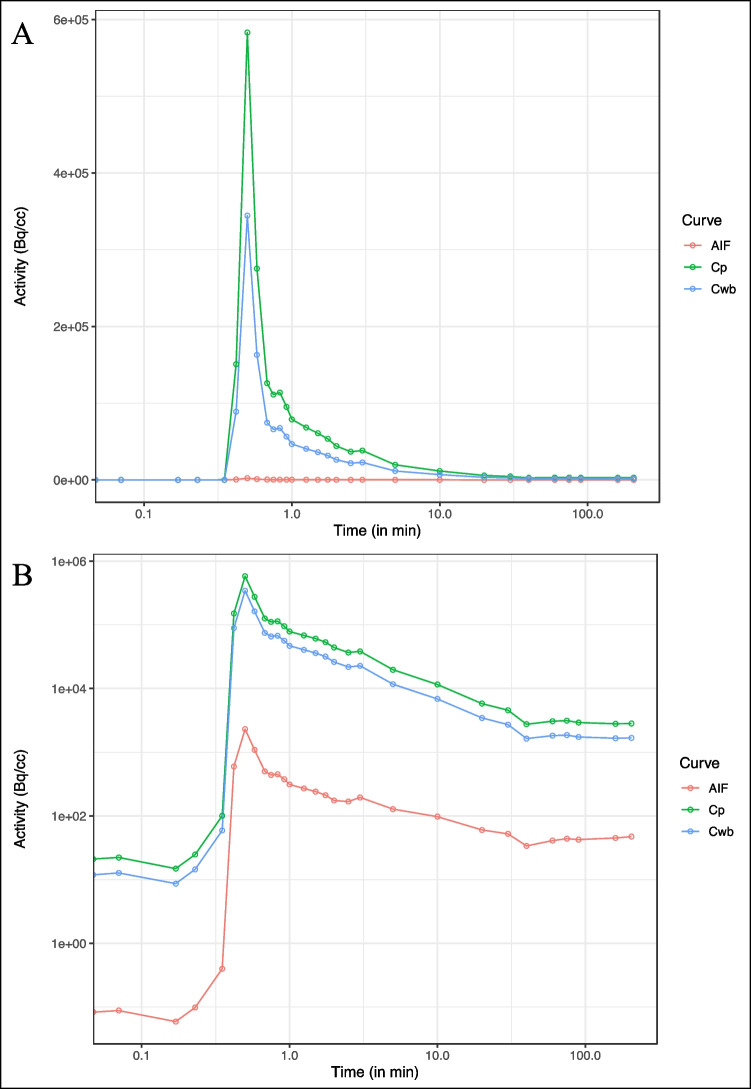
Example of an arterial input function (AIF) for a subject with whole blood (Cwb) and uncorrected plasma curve (Cp). **A** Logarithmic time scale. **B** Double-logarithmic scale for the same subject

Free plasmatic fraction was not constant over time and mean values were 0.48% ± 0.09%, 0.82% ± 0.08%, 1.14% ± 0.26%, 1.90% ± 0.71% and 1.61% ± 0.29% at 2, 10, 30, 90, and 205 min. Ratio plasma to whole blood was stable over time and mean value was 1.79 ± 0.03. Table 2 summarizes the pharmacokinetic parameters for each subject. Figure 2 shows an example of AIF corrected for metabolites, whole blood, and uncorrected plasma curves. Individual TACs and AIF are presented in online resource 1 (Fig S1, S2, S3, and S4).

**Table 2:** Details of pharmacokinetics parameters per subject. Plasma parent fraction and free plasma fraction results are expressed as a function of time. Plasma to whole blood was stable over time and results are expressed as a mean value across the sessions for each participant

Parameter	Plasma parent fraction (%)					Free plasma fraction (%)					Plasma to whole blood ratio
^Time (min)^	2	10	30	90	205	2	10	30	90	205	Mean value ± SD
_Subject_											
2	99.5	96.1	95.4	NA	96.7	0.42	0.72	1.10	1.23	1.94	1.84 ± 0.11
5	99.5	97.9	97.8	96.7	95.6	0.52	0.79	0.88	2.87	1.66	1.78 ± 0.11
7	98.9	97.5	95.8	94.9	94.7	0.38	0.88	1.08	1.56	1.62	1.77 ± 0.14
8	99.5	98.0	95.6	94.5	94.3	0.59	0.89	1.51	1.94	1.24	1.77 ± 0.14
*Mean* ± *SD*	*99.40 *± *0.00*	*97.40 * ± *0.01*	*96.20 * ± *0.01*	*95.40 *± *0.01*	*95.30 * ± *0.01*	*0.48 * ± *0.09*	*0.82 * ± *0.08*	*1.14 * ± *0.26*	*1.90 *± *0.71*	*1.61 * ± *0.29*	*1.79* ± *0.03*

Mean AIC using the one-tissue compartment model (1TC) was 320.88 ± 13.66 and 291.92 ± 25.46 for the two-tissue compartment model (2TC). Two-tissue compartment model better fits with the pharmacokinetic of [^18^F]F13640 when comparing the AIC but results in nonsensical numerical kinetics parameters (k_3_ and k_4_). Thus, the 1TC model was considered as the reference model for the results presented below. Fitted curves for 1TC and 2TC modeling are presented in online resource 1 (Fig S5 and S6).

Pharmacokinetic parameters for the 1TC model are presented in Table 3. The highest Vt values were found in raphe nuclei (median and dorsal), cingulate, amygdala, and insula. The lowest Vt values were in cerebellum white matter, frontal lobe white matter, and corpus callosum, all tested as reference regions. The cerebellum (grey + white matter), also tested as a reference region, showed intermediate Vt values.

**Table 3:** One-tissue compartment model parameters for 4 subjects (Vt: total volume distribution, Vb: blood volume). Data are presented as mean ± SD

Regions	K1 (mL/(min*mL))	k2 (min^−1^)	Vt (mL/mL)	Vb (%)
*Cortical region*				
Cingulate lobe	1.38 ± 0.31	0.014 ± 0.004	98.4 ± 19.6	3.8 ± 0.4
Frontal lobe	1.33 ± 0.24	0.016 ± 0.004	86.9 ± 19.4	3.3 ± 0.3
Occipital lobe	1.26 ± 0.28	0.017 ± 0.004	77.9 ± 17.5	3.6 ± 0.4
Parietal lobe	1.32 ± 0.33	0.016 ± 0.004	83.6 ± 19.4	3.8 ± 0.3
Temporal superior lobe	1.31 ± 0.29	0.016 ± 0.004	85.5 ± 20.7	4.0 ± 0.8
Temporal inferior lobe	1.19 ± 0.29	0.016 ± 0.004	78.2 ± 20.0	2.9 ± 0.4
*Subcortical region*				
Amygdala	1.07 ± 0.22	0.013 ± 0.003	87.8 ± 20.2	3.6 ± 0.6
Central grey nuclei	1.07 ± 0.27	0.016 ± 0.004	71.3 ± 18.5	3.0 ± 0.5
Hippocampus	1.04 ± 0.25	0.013 ± 0.003	83.3 ± 19.8	3.5 ± 0.6
Insula	1.18 ± 0.26	0.014 ± 0.004	88.1 ± 21.3	3.7 ± 0.4
Parahippocampal gyrus	1.05 ± 0.21	0.014 ± 0.004	75.8 ± 15.9	4.4 ± 0.8
Thalamus	1.18 ± 0.28	0.015 ± 0.004	84.4 ± 21.6	3.8 ± 0.3
*Brainstem*				
Brainstem	1.03 ± 0.26	0.013 ± 0.003	81.6 ± 21.6	3.3 ± 0.4
Dorsal raphe nucleus	1.21 ± 0.33	0.013 ± 0.004	96.6 ± 28.4	3.3 ± 0.5
Median raphe nucleus	1.13 ± 0.31	0.012 ± 0.003	93.8 ± 26.0	3.2 ± 0.5
*Cerebellum*				
Cerebellum	1.38 ± 0.37	0.016 ± 0.004	86.6 ± 20.4	4.2 ± 0.4
Cerebellum grey matter	1.38 ± 0.41	0.016 ± 0.004	86.6 ± 22.0	4.1 ± 0.4
Cerebellum white matter	1.03 ± 0.25	0.015 ± 0.003	68.7 ± 16.9	2.8 ± 0.3
Vermis	1.41 ± 0.40	0.017 ± 0.004	86.7 ± 22.0	3.9 ± 0.4
*Reference region*				
Frontal lobe white matter	0.81 ± 0.16	0.013 ± 0.004	63.3 ± 13.7	2.0 ± 0.3
Corpus callosum	0.56 ± 0.11	0.011 ± 0.003	49.8 ± 9.9	2.2 ± 0.4

Linear regressions of DVR_LREF_ and DVR_SRTM_, compared to DVR_1TC_ with the four reference regions tested, are presented in Table 4. For LREF model, all coefficients of determination (*R*^2^) were very high (> 0.9). Best fitting was obtained with LREF_CERWM_ and LREF_CER_ (0.95 ± 0.03 both). Note that both LREF_CC_ and LREF_FLWM_ showed high *R*^2^ values (0.94 ± 0.03 and 0.94 ± 0.04, respectively). Intercept ranged from 0.01 ± 0.05 (LREF_CER_) to 0.04 ± 0.08 (LREF_CC_) thus best fitting (closest to 0) was obtained with LREF_CER_. Slopes ranged from 0.93 ± 0.02 (LREF_CC_) to 1.01 ± 0.03 (LREF_CERWM_) and the best slope value was found with LREF_CERWM_ (1.01 ± 0.02). For the SRTM model, regression parameters (*R*^2^) were slightly lower than those found with LREF but close to 0.9, except for CC which showed the lowest *R*^2^ value (0.59 ± 0.10). Intercepts ranged from 0.04 ± 0.09 for SRTM_CER_ (best fitting) to 0.68 ± 0.12 for SRTM_CC_. Slopes ranged from 0.67 ± 0.13 (SRTM_CC_) to 0.93 ± 0.13 (SRTM_CER_). SRTM_CERWM_ and SRTM_FLWM_ showed acceptable slopes with 0.92 ± 0.09 and 0.91 ± 0.10, respectively.

**Table 4:** Regression parameter of DVR_SRTM_ and DVR_LREF_ compared to DVR_1TC_ for 4 subjects with AIF. Test–retest parameters for reproducibility assessment in 8 healthy subjects. Reference region tested were corpus callosum (CC), cerebellum (CER), cerebellum white matter (CERWM), and frontal lobe white matter (FLWM). Data are presented as mean ± SD. ICC = interclass correlation coefficient, VAR = variability

	Mean regression parameter compared to DVR_1TC_				Mean test–retest parameters		
Model	Reference Region	Slope	Intercept	R^2^	ICC	Bias (%)	VAR (%)
DVR_LREF_	CC	0.93 ± 0.02	0.04 ± 0.08	0.94 ± 0.03	0.89 ± 0.10	0.95	2.84
	CER	1.01 ± 0.03	0.01 ± 0.05	0.95 ± 0.03	0.93 ± 0.13	− 0.27	2.81
	CERWM	1.01 ± 0.02	0.02 ± 0.06	0.95 ± 0.03	0.95 ± 0.04	− 0.04	2.41
	FLWM	0.97 ± 0.03	0.03 ± 0.08	0.94 ± 0.04	0.88 ± 0.08	0.24	2.55
DVR_SRTM_	CC	0.67 ± 0.13	0.68 ± 0.12	0.59 ± 0.10	0.67 ± 0.17	0.23	11.34
	CER	0.93 ± 0.13	0.04 ± 0.09	0.90 ± 0.03	0.75 ± 0.26	− 0.55	7.16
	CERWM	0.92 ± 0.09	0.10 ± 0.06	0.89 ± 0.03	0.63 ± 0.23	− 1.48	8.76
	FLWM	0.91 ± 0.10	0.14 ± 0.09	0.90 ± 0.10	0.47 ± 0.31	0.34	8.51

**Fig. 3:**
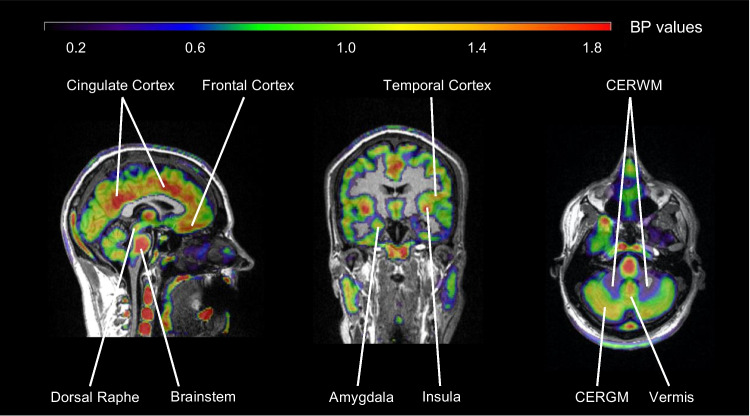
Parametric image of BP values estimated with LREF_CERWM_. BP image is overlaid on the T1 MRI of the participant (CERWM = cerebellum white matter; CERGM = cerebellum grey matter)

Parametric images were obtained using models with reference region. As an example, Fig. 3 shows a brain parametric image of binding potential (BP) using the LREF modeling method with CERWM as reference region.

### Test–retest reproducibility and reliability

Test–retest study results are presented in Table 4. Reproducibility was high for the LREF method and satisfying for SRTM. Biases were similar between modeling methods and between reference regions. Results ranged from − 1.48 to 0.95% with the lowest bias for LREF_CERWM_ (-0.04%). As concerns variability (VAR), results were more disparate between methods. The LREF method showed a very low variability of less than 3% for all reference regions. The lowest VAR was found for LREF_CEWRM_ with 2.41%. VAR values for other reference regions were also very low with 2.55%, 2.81%, and 2.84% for LREF_FLWM_, LREF_CER_, LREF_CC,_ respectively. SRTM showed higher values. The lowest VAR was SRTM_CER_ with 7.16%. Variability for SRTM_CERWM_ and SRTM_FLWM_ was also satisfying with 8.76% and 8.51%, respectively. Reliability showed better results for the LREF method than with SRTM, with all ICC around 0.9 and the best performance for LREF_CERWM_ (0.95 ± 0.04). The STRM method showed ICC values that were lower and with spread over a wider range from 0.47 ± 0.31 for SRTM_FLWM_ to 0.75 ± 0.26 for SRTM_CER_. ICCs for SRTM_CERWM_ and SRTM_CC_ were intermediate with 0.63 ± 0.23 and 0.67 ± 0.17, respectively.

Table 5 summarizes reproducibility and reliability parameters in each region for SRTM_CERWM_ and LREF_CERWM_. DVR was highly correlated between test and retest whatever the method used (*R*^2^ > 0.99 for LREF_CERWM_ and *R*^2^ > 0.98 for SRTM_CERWM_). ICC, bias, and VAR per region confirmed the results described in Table 4 and showed that the overall best parameters were those observed using the LREF_CERWM_ method.

**Table 5:** Reliability and reproducibility parameters between test and retest per region for Logan graphical method (LREF) and Simplified Reference Tissue Model (SRTM) using white matter cerebellum as reference region (CERWM). Parameters calculated were interclass correlation coefficient (ICC), variability (VAR), and bias. DVRs are expressed as mean ± SD. (CERGM = cerebellum grey matter; FLWM = frontal lobe white matter)

Model	Region	DVR test	DVR retest	ICC	Bias (%)	VAR (%)
LREF_CERWM_	*Cortical region*					
	Cingulate	1.38 ± 0.11	1.37 ± 0,10	0.97	0.31	1.78
	Frontal	1.28 ± 0.09	1.28 ± 0,09	0.97	0.54	1.80
	Occipital	1.06 ± 0.10	1.06 ± 0,09	0.99	− 0.24	1.64
	Parietal	1.20 ± 0.10	1.20 ± 0,09	0.97	− 0.30	2.16
	Temporal inferior	1.15 ± 0.10	1.15 ± 0,09	0.92	− 0.01	3.42
	Temporal superior	1.24 ± 0.09	1.24 ± 0,09	0.93	− 0.19	2.95
	*Subcortical region*					
	Amygdala	1.20 ± 0.07	1.19 ± 0,06	0.90	0.07	2.28
	Central grey nuclei	1.03 ± 0.16	1.03 ± 0,15	0.94	0.38	2.30
	Hippocampus	1.15 ± 0.07	1.15 ± 0,06	0.92	− 0.37	2.10
	Insula	1.22 ± 0.08	1.21 ± 0,08	0.94	0.55	2.42
	Parahippocampal gyrus	1.08 ± 0.08	1.09 ± 0,07	0.95	− 0.79	2.47
	Thalamus	1.16 ± 0.09	1.15 ± 0,08	0.99	0.48	1.20
	*Brainstem*					
	Brainstem	1.12 ± 0.09	1.12 ± 0.08	0.99	0.25	1.09
	Dorsal raphe nucleus	1.33 ± 0.12	1.33 ± 0.11	0.94	0.25	3.12
	Median raphe nucleus	1.26 ± 0.12	1.27 ± 0.14	0.81	− 1.27	6.62
	Cerebellum					
	Cerebellum	1.31 ± 0.07	1.31 ± 0.07	0.96	0.23	1.50
	CERGM	1.31 ± 0.07	1.31 ± 0.07	0.96	0.23	1.50
	Vermis	1.32 ± 0.07	1.32 ± 0.07	0.98	0.20	0.92
	*Reference region*					
	FLWM	0.89 ± 0.06	0.89 ± 0.05	0.97	− 0.31	1.66
	Corpus callosum	0.68 ± 0.06	0.68 ± 0.04	0.92	− 0.93	2.84
SRTM_CERWM_	*Cortical region*					
	Cingulate	1.36 ± 0.15	1.40 ± 0.14	0.74	− 3.04	7.09
	Frontal	1.18 ± 0.16	1.23 ± 0.15	0.86	− 4.33	6.26
	Occipital	0.97 ± 0.13	0.99 ± 0.13	0.69	− 1.49	11.95
	Parietal	1.11 ± 0.14	1.13 ± 0.12	0.70	− 2.36	10.28
	Temporal inferior	1.09 ± 0.17	1.11 ± 0.10	0.62	− 2.72	11.15
	Temporal superior	1.18 ± 0.19	1.21 ± 0.13	0.48	− 3.04	14.74
	*Subcortical region*					
	Amygdala	1.30 ± 0.14	1.30 ± 0.12	0.65	0.08	7.90
	Central grey nuclei	0.99 ± 0.18	1.01 ± 0.17	0.77	− 1.96	6.84
	Hippocampus	1.24 ± 0.11	1.20 ± 0.12	0.62	3.11	7.34
	Insula	1.24 ± 0.15	1.26 ± 0.14	0.73	− 1.58	8.55
	Parahippocampal gyrus	1.08 ± 0.08	1.08 ± 0.07	0.95	0.18	2.49
	Thalamus	1.15 ± 0.15	1.16 ± 0.12	0.79	− 1.50	7.75
	*Brainstem*					
	Brainstem	1.21 ± 0.13	1.21 ± 0.15	0.88	0.30	6.54
	Dorsal raphe nucleus	1.41 ± 0.11	1.42 ± 0.17	0.55	− 0.69	9.70
	Median raphe nucleus	1.36 ± 0.21	1.39 ± 0.18	− 0.07	− 2.23	20.03
	*Cerebellum*					
	Cerebellum	1.25 ± 0.07	1.26 ± 0.06	0.33	− 0.93	5.60
	CERGM	1.25 ± 0.06	1.27 ± 0.05	0.35	− 1.83	5.17
	Vermis	1.23 ± 0.07	1.24 ± 0.08	0.74	− 1.12	4.46
	*Reference region*					
	Corpus callosum	0.77 ± 0.11	0.80 ± 0.08	0.63	− 2.97	10.65
	FLWM	0.90 ± 0.07	0.92 ± 0.10	0.58	− 2.92	7.61

Considering LREF_CERWM_, bias was consistently low, between − 1 and 1%, for all regions. Only the median raphe nucleus showed bias outside this range (− 1.27%). Variability was very low for all regions, with values around or less than 3%, except for the median raphe nucleus (6.62%). Reliability was high (> 0.7) for all regions. Considering SRTM_CERWM_, biases were all satisfying, with higher values observed for the frontal cortex (− 4.33%) and hippocampus (3.11%). For variability, the median raphe nucleus showed the highest value (20.03%). The temporal (superior and inferior), occipital, parietal cortex, and corpus callosum showed VAR higher than 10%. Other regions showed variability of less than 10%. Reliability was low for the median raphe nucleus (− 0.07), cerebellum (0.33), and cerebellum grey matter (CERGM = 0.35). Some regions showed particularly high reliability with ICC values above 0.7, such as the cingulate (0.74) and frontal cortex (0.86), and the highest ICC value was found for the parahippocampal gyrus (0.95). Other regions showed intermediate ICC from 0.5 to 0.7 such as the hippocampus (0.62) and amygdala (0.65).

## Discussion

In this study, we describe the first-in-human trial of a new PET radiopharmaceutical [^18^F]F13640 which, thanks to its agonist properties, permits in vivo imaging of functional human 5-HT_1A_ receptors. Several analyses were carried out: (i) full kinetic modeling of the radiopharmaceutical using dynamic scans and arterial blood sampling; (ii) comparison of four different reference regions and evaluation of two simplified modeling methods to measure binding potential values and obtain parametric images; and (iii) assessment of test–retest reliability for simplified models. The main finding is that [^18^F]F13640 constitutes a 5-HT_1A_ receptor radiopharmaceutical with favorable brain binding properties, and the present study describes a PET acquisition protocol and a quantification method suitable for the clinical study of functional 5-HT_1A_ receptors in neurology and psychiatry.

### Study design

Initial tests used a protocol with 90 min PET/CT scans and AIF for study participants. However, preliminary analyses of the 5 first subjects revealed a slow washout from cerebral tissues of the radiopharmaceutical, a characteristic that has also been observed for some other radiopharmaceuticals, such as [^18^F]fallypride [33]. This observation is in accordance with our preclinical observations in rat, cat, and macaque [21]. Since [^18^F]F13640 has a high affinity for the target sites, the low tissue clearance k2 observed on kinetics could be interpreted as a very low dissociation rate on 5-HT_1A_ receptors. On the basis of these preliminary observations, we concluded that kinetic modeling of the tracer and parameters identification would require a longer acquisition protocol, which was then implemented for the main study. A 165-min long PET acquisition was implemented with a first scan lasting 90 min and a second one lasting 75 min, separated by a 1-h break outside of the camera. This longer protocol with two acquisition periods was facilitated using a hybrid PET-MR scanner, which is capable of brain anatomical realignment and motion correction between PET acquisitions.

### Modeling study

Our data revealed that the pharmacokinetic parameters of [^18^F]F13640 were similar to those found with F13640 (i.e., NLX-112, befiradol) at pharmacological doses. Thus, the fraction of [^18^F]F13640 parent compound remained high (95%) in blood at 30 to 205 min after injection, consistent with observations on unlabeled NLX-112 in healthy volunteers (Neurolixis, data on file). The ratio of [^18^F]F13640 binding in plasma to whole blood showed favorable results with a higher distribution in plasma (1.79 ± 0.03). Also, the *fp* of about 2% was similar between NLX-112 and [^18^F]F13640. However, we observed unreasonably high *K*_1_ values since they were higher than cerebral blood flow investigated with [^15^O]H_2_O PET [1, 2]. A plausible, but not a necessarily unique explanation, is that *fp* measurement has a highly variable value in the first few minutes, and stabilizes later towards a very low value. Processing of the blood samples required 20 to 25 min before being measured for protein binding, and thus may not represent the “true” *fp* at an early time of acquisition. Therefore, *K*_1_ estimates are prone to inaccuracy since *fp* may not have reached its low equilibrium at short time points after injection. Based on this observation, we also performed 1TC modeling using only mean late-time *fp* value since they were considered to be more reliable and in accordance with data generated using unlabeled F13640 (Neurolixis, data on file). As expected, this modeling study showed more realistic values (see table S1 in online resource). It should be noted that the calculation of *K*_1_ does not constitute an obstacle for use of [^18^F]F13640 because data generated using this radiopharmaceutical is intended to be analyzed with a simplified method or under static scans with reference region and thus will not require *fp* value.

A crucial step in the validation of a new brain receptor radiopharmaceutical is the validation of a reference region. Based on literature describing regions poor in 5-HT_1A_ receptors, we tested four different reference regions: corpus callosum (CC), cerebellum (CER), cerebellum white matter (CERWM), and frontal lobe white matter (FLWM). We tested their accuracy and reproducibility performance with two simplified modeling methods, the LREF and SRTM. For the validation with a gold standard, very favorable correlations have been found with the LREF model compared to the 1TC AIF, whatever the reference region. The SRTM method also showed satisfying results in regression whereas compared to 1TC AIF model, except for CC as reference, showed the weakest results. For test–retest reproducibility, ICC, bias, and variability were similar for LREF with the four reference regions, with superior performance when using CERWM as reference region. With SRTM methods, performance for reliability is more mixed, with a clear superiority for the CERWM reference region. Some assumptions need to be validated for the use of SRTM: reference region has no specific binding, the K_1_/k_2_ parameter needs to be the same in the reference region, and regions of interest and kinetics can be fitted with the 1TC model. All these assumptions were confirmed with [^18^F]F13640 since CERWM is known to have very low 5-HT_1A_ receptors expression [35], the K_1_/k_2_ parameter was similar between CERWM and regions of interest such as hippocampus or cingulate, and CERWM was fitted with the 1TC model. Taken together, these results indicate that CERWM is the best reference region, showing a low binding, a high correlation with the AIF model, and favorable test–retest reproducibility. Both the LREF and SRTM methods showed similar binding values for all brain regions. Using the SRTM method, the amygdala and hippocampus showed high BP while LREF-calculated BP values were intermediate. According to the 1TC model, the binding values of the amygdala and hippocampus calculated with SRTM are more accurate than the one with LREF. However, considering test–retest parameters, better reproducibility and reliability were found with the LREF method. The high level of test–retest reliability is confirmed at the regional level for LREF with the CERWM, something which is not systematically the case for SRTM and CERWM.

Overall, these results lead us to favor the LREF model with CERWM as reference region for future studies comparing patients to healthy volunteers or patients at different times. The low variability and high reliability (ICC > 0.7) of the LREF model with CERWM as reference region guarantee the power of future studies and the capacity to detect differences between groups due to pathological changes rather than inter-participant or protocol variability. However, the SRTM modeling method and FLWM as an alternative reference region are not to be totally discarded. Indeed, the choice of the modeling method and of the reference region will be driven by the pathology in the study. As an example, cerebellar atrophy could be a confounding factor when using CERWM as a reference region and FLWM would be a suitable alternative. In the present study, the LREF method with CERWM as reference region was used to evaluate [^18^F]F13640 binding patterns, as discussed below.

Finally, modeling was also computed with the first 90 min (online resource 1 Fig S7). Correlations of Vt by subject, evaluated with 90 min versus 225 min were acceptable but somewhat lower than for shorter scans (*R*^2^ between 0.55 to 0.75), with a slight bias (slopes between 0.66 and 1.05) and small intercepts (0.22–0.61). Performing scans of 90 min are therefore feasible but not optimal for an accurate evaluation of the binding potential.

### [^18^F]F13640 brain binding patterns

[^18^F]F13640 showed elevated BP values in regions known to express a high density of 5-HT_1A_ receptors, including the raphe (median and dorsal) and cortical regions (cingulate, frontal, and temporal superior) [36]. It is noteworthy that the hippocampus only showed intermediate uptake of [^18^F]F13640 with moderate BP values. Median and dorsal raphe nuclei were regions with the highest BP values but also with the most substantial variability. This can be explained by the fact that the raphe nucleus is the only region that is not defined on the basis of anatomical data but by functional data [29]. Note that some vessels showed also high binding value and could affect the BP of the nearest regions due to a partial volume effect.

### [^18^F]F13640 binding in cerebellum grey matter

As described above, cerebellum grey matter and vermis both showed high BP values. Whereas a high expression of 5-HT_1A_ receptors has been described in vermis [35, 37], it is frequently postulated that cerebellum grey matter does not express 5-HT_1A_ receptors. Indeed, according to post-mortem autoradiographic studies [38, 39], 5-HT_1A_ receptors are expressed in fetal and neonatal stages but not in adults [40]. The present unexpected binding of [^18^F]F13640 in the cerebellum could thus question its target specificity, but an interaction by the radiotracer with a possible off-target seems unlikely based on well-documented in vitro studies on F13640 [20]. Moreover, our previous preclinical studies also detected [^18^F]F13640 binding in the cerebellum in different species [21, 22] and, in all these studies, co-administration of pharmacologically relevant doses of a 5-HT_1A_ receptor agonist (8-OH-DPAT) or of an antagonist (WAY-100635) inhibited [^18^F]F13640 binding, indicating that its binding in cerebellum grey matter specifically reflects 5-HT_1A_ receptor expression in this region. In addition to the present data with [^18^F]F13640, the assumption of a total lack of 5-HT_1A_ receptors in the cerebellum was already challenged by previous PET studies revealing [*carbonyl-*^11^C]WAY-100635 binding in human cerebellum [35], with more pronounced labeling in grey matter [37]. In other studies, participants were excluded due to cerebellar TACs outside the range of the control population when the cerebellum was used as a reference region [41–43]. Other 5-HT_1A_ receptor radiopharmaceuticals also showed marked binding in the cerebellum, notably [^11^C]CUMI-101 which showed a significant reduction of labeling following 8-OH-DPAT or WAY-100635 pre-injection [44]. Taken together, these observations indicate that 5-HT_1A_ receptors are indeed present in cerebellum grey matter, excluding the cerebellum (which contain the grey matter) as a reference region for [^18^F]F13640.

### [^18^F]F13640 binding in other brain regions

In comparison with other 5-HT_1A_ radiopharmaceuticals, [^18^F]F13640 showed particularly high uptake in raphe nuclei, cortical regions, and vermis in limbic and paralimbic regions. Although [^18^F]MPPF also showed high uptake in raphe nuclei, it preferentially binds to limbic areas such as the hippocampus and amygdala whereas cortical regions showed less uptake [45]. Another antagonist radiopharmaceutical, [*carbonyl-*^11^C]WAY-100635, also showed high binding in the raphe and frontal cortex (like [^18^F]F13640) but it also showed marked binding to the hippocampus (like [^18^F]MPPF) [3]. Binding differences between [*carbonyl-*^11^C]WAY-100635, [^18^F]MPPF and [^18^F]F13640 are likely to be due to pharmacological differences between these 5-HT_1A_ receptor radiopharmaceuticals [46, 47]. We hypothesize that some regions such as the hippocampus, which showed high uptake with antagonist radiopharmaceuticals and less with the agonist [^18^F]F13640, express a high proportion of G-protein uncoupled 5-HT_1A_ receptors. In contrast, regions with higher [^18^F]F13640 binding values, such as the cingulate cortex, frontal cortex, or vermis, may express a relatively higher proportion of functionally active, G-protein-coupled receptors. Finally, regions such as raphe which showed high binding with both antagonist and agonist radiopharmaceuticals may express both states of 5-HT_1A_ receptors. Thus, differences in receptor binding patterns can be found between agonists and antagonists radiopharmaceuticals targeting the same receptor. An in vitro study using [^18^F]F13640 and [^18^F]MPPF already explored this hypothesis and showed differences in binding between agonists and antagonists radiotracer with 5-HT_1A_ in Alzheimer’s disease [48]. Analogous results were found with D_2/3_ dopamine receptors, for which the agonist, [^3^H]NPA, showed a greater extent of than the antagonist [^11^C]raclopride binding in a rat Parkinson’s disease model [49].

### Perspectives opened by [^18^F]F13640

Based on the above observations, it can be considered that [^18^F]F13640 has now the status of a radiopharmaceutical whose novel pharmacokinetic and radiopharmacological characteristics offer new perspectives in neurology and psychiatry.

First of all, the sustained binding of [^18^F]F13640 will allow studies to be performed with multiple acquisitions at different late time points following the same injection. This experimental paradigm could, for example, allow exploration of changes in receptor coupling states associated with circadian rhythms. As an illustration, a previous preclinical study demonstrated that [^18^F]F13640 is sensitive to fluctuations in serotonin levels [22] opening the way for exploration of in vivo serotonin release in physiological or pathological processes.

As demonstrated in the present study, [^18^F]F13640 binding using the LREF method showed excellent test–retest parameters, a profile which makes it attractive for studies involving repeated measurements on the same subject, including drug occupancy and intervention studies, or for exploring pathological states at different time points—situations where reproducibility and reliability of the PET measurements is crucial.

In terms of its radiopharmacological characteristics, the binding pattern of [^18^F]F13640 opens the way to informative imaging studies in neurology and psychiatry. For example, [^18^F]F13640 shows high binding values in the raphe, consistent with the therapeutic-like activity of 5-HT_1A_ receptor agonists in rodent and non-human primate models of L-DOPA-induced dyskinesia (LID) in Parkinson’s disease [50–52] which is the focus of an ongoing clinical study (ClinicalTrials.gov Identifier: NCT05148884). [^18^F]F13640 also shows high binding values in cortical regions associated with antidepressant activity or control of negative symptoms of schizophrenia [1–3]. 5-HT_1A_ receptors in the brainstem are also promising targets to alleviate respiratory dysfunction in disorders such as Rett syndrome [56]. [^18^F]F13640 could therefore be a promising tool to assess the role of 5-HT_1A_ receptors in a variety of different disorders involving serotonergic mechanisms [15].

## Conclusion

The present study reports the first-in-human validation and full kinetic modeling of [^18^F]F13640 as the first 5-HT_1A_ receptor agonist usable as a PET radiopharmaceutical. [^18^F]F13640 shows many favorable radiopharmacological and radiopharmaceutical characteristics: radiolabeling with fluorine-18, high selectivity over cross-reacting sites, high reproducibility, and long-term binding which facilitates the experimental protocols. [^18^F]F13640 shows pronounced binding in raphe nuclei and cortical regions, with notable differences in comparison with classical antagonist PET radiopharmaceuticals. All these characteristics confirm the interest of developing an agonist radiotracer able to specifically target functionally active 5-HT_1A_ receptors in studies with long-term scans and test–retest protocols in order to investigate disease states in neurology and psychiatry.

## Supplementary Information

Below is the link to the electronic supplementary material.

Supplementary file1 (PDF 453 KB)

## Data Availability

The datasets generated during this study are available from the corresponding author.
