# Supplementary material for: [18F]F13640: a selective agonist PET radiopharmaceutical for imaging functional 5-HT1A receptors in humans
Source: Eur J Nucl Med Mol Imaging. 2023 Jan 19;50(6):1651–64. doi: 10.1007/s00259-022-06103-1 (PMC10119077; doi:10.1007/s00259-022-06103-1)
Supplement: Supplementary file 1 — Supplementary file1 (PDF 453 KB) [file 259_2022_6103_MOESM1_ESM.pdf]

Supplementary Online resource 1

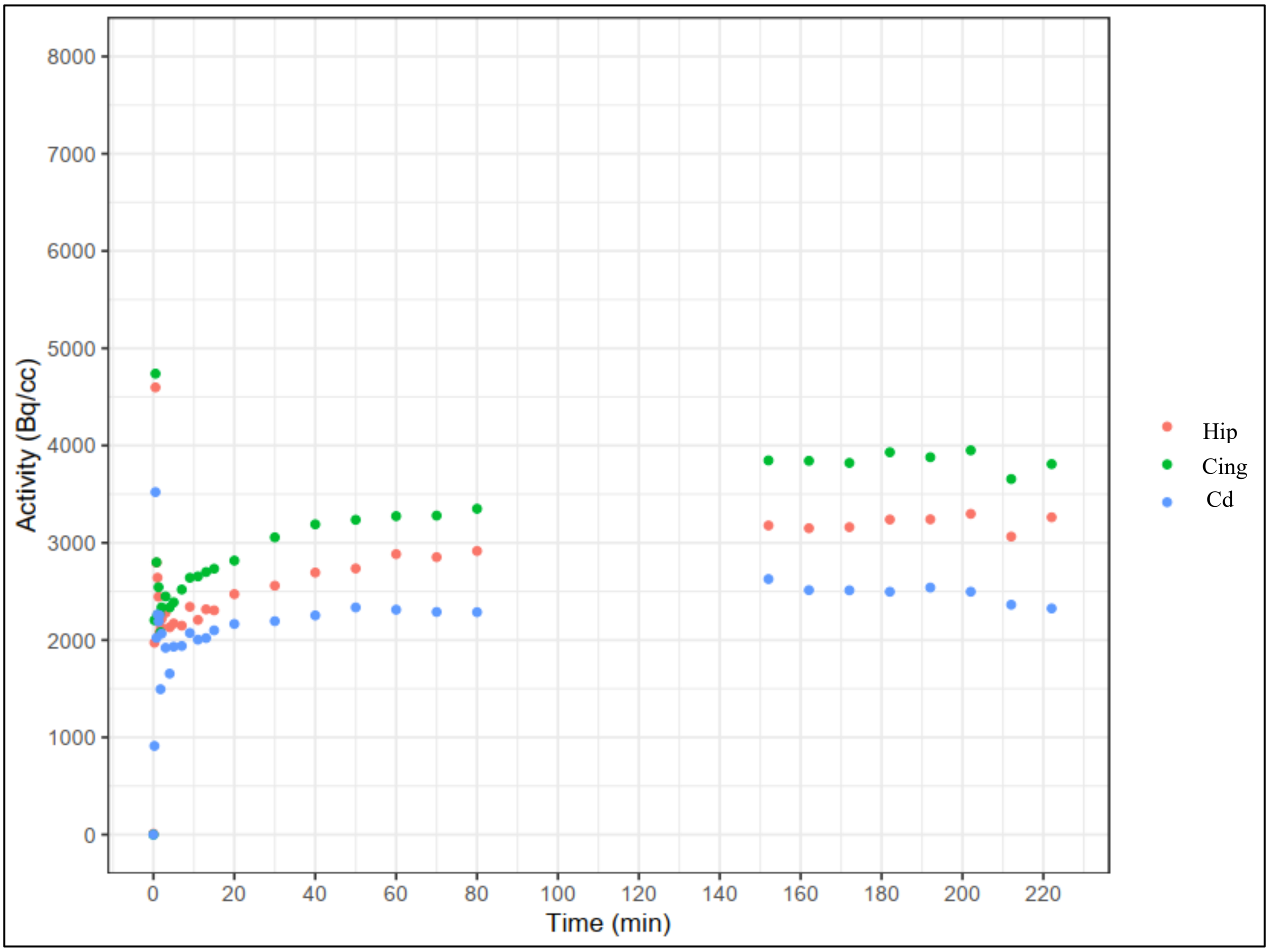

Figure S1. Example of 225 min time activity curves of three different regions (Hip: Hippocampus; Cing: Cingulate; Cd: Caudate) for subject 2 of the PET-MRI study.

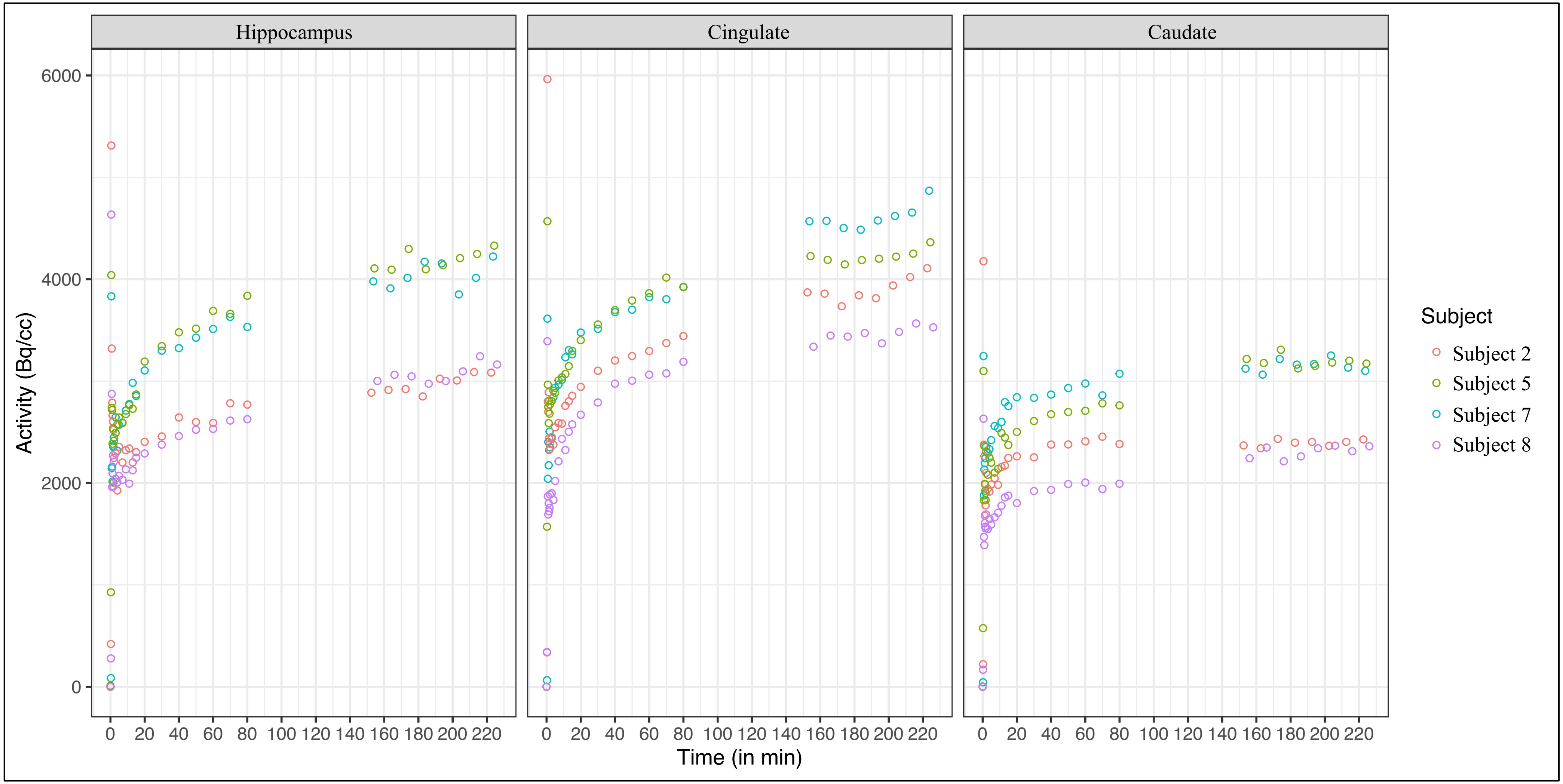

Figure S2. Inter-subject variability of time activity curves in three different regions.

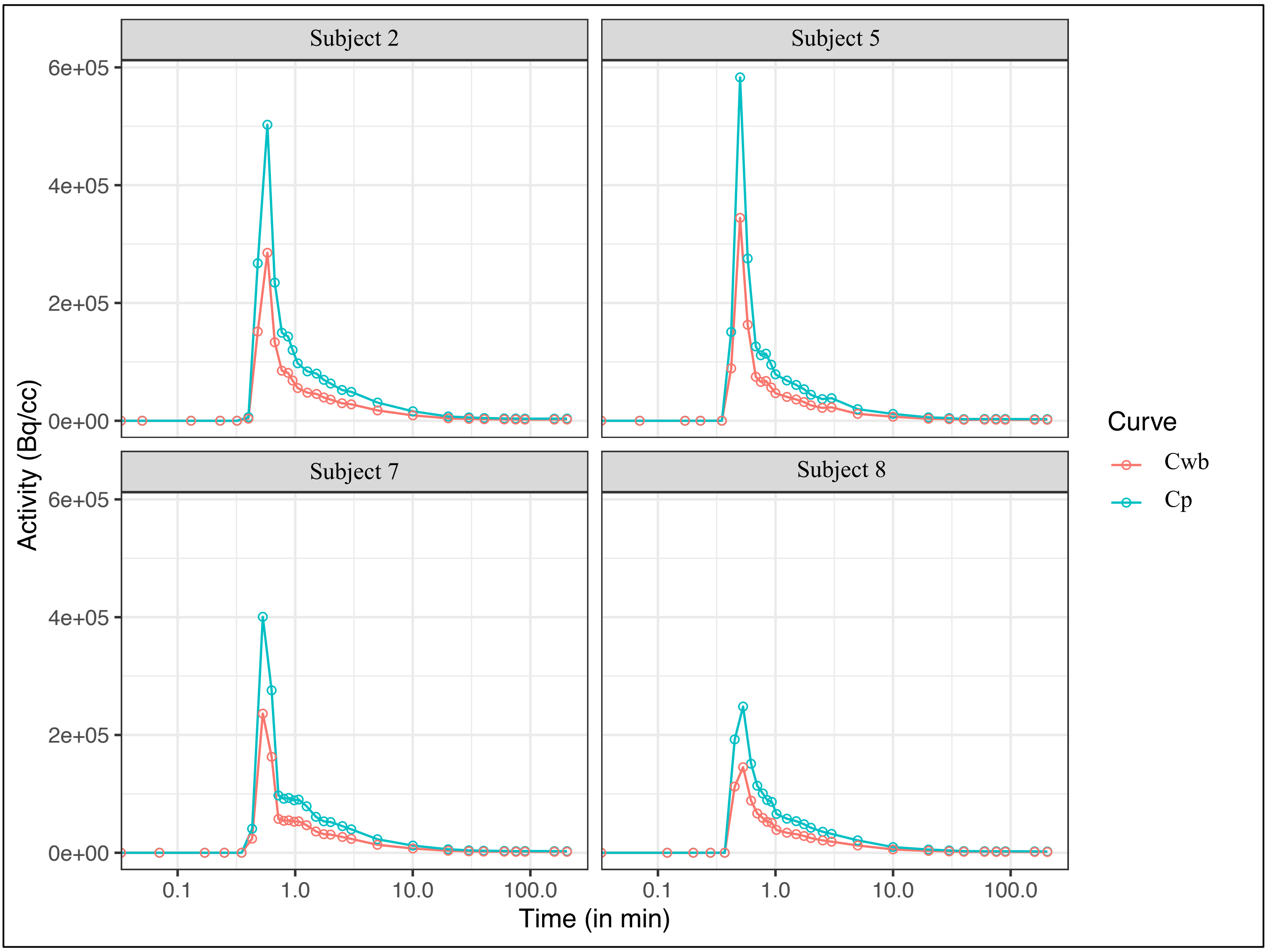

Figure S3. Logarithmic time scale, scale, of arterial input function (AIF) for the four patients, with whole blood (Cwb) and uncorrected plasma curve (Cp).

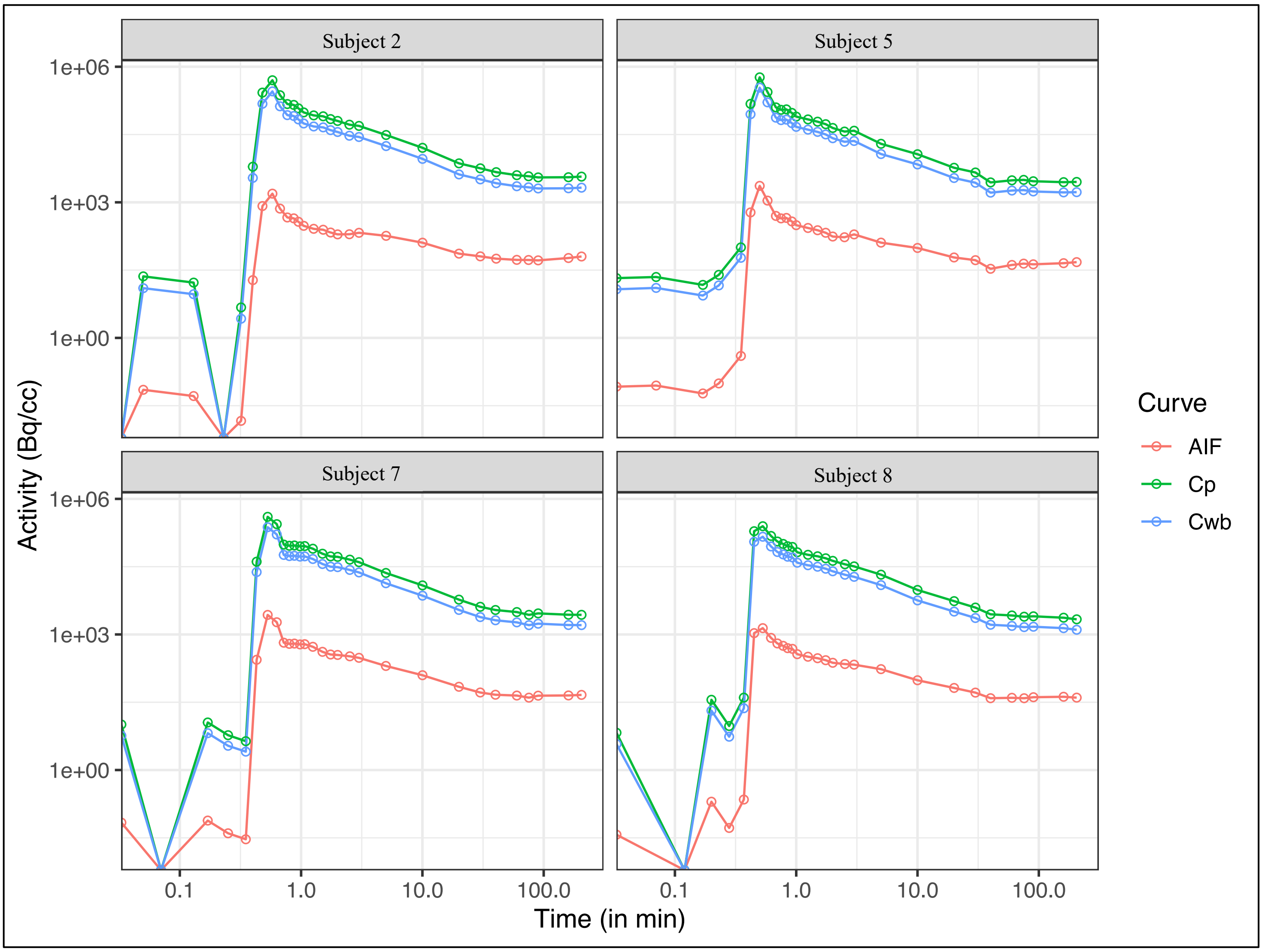

Figure S4. Double-logarithmic scale of arterial input function (AIF) for the four patients, with whole blood (Cwb) and uncorrected plasma curve (Cp).

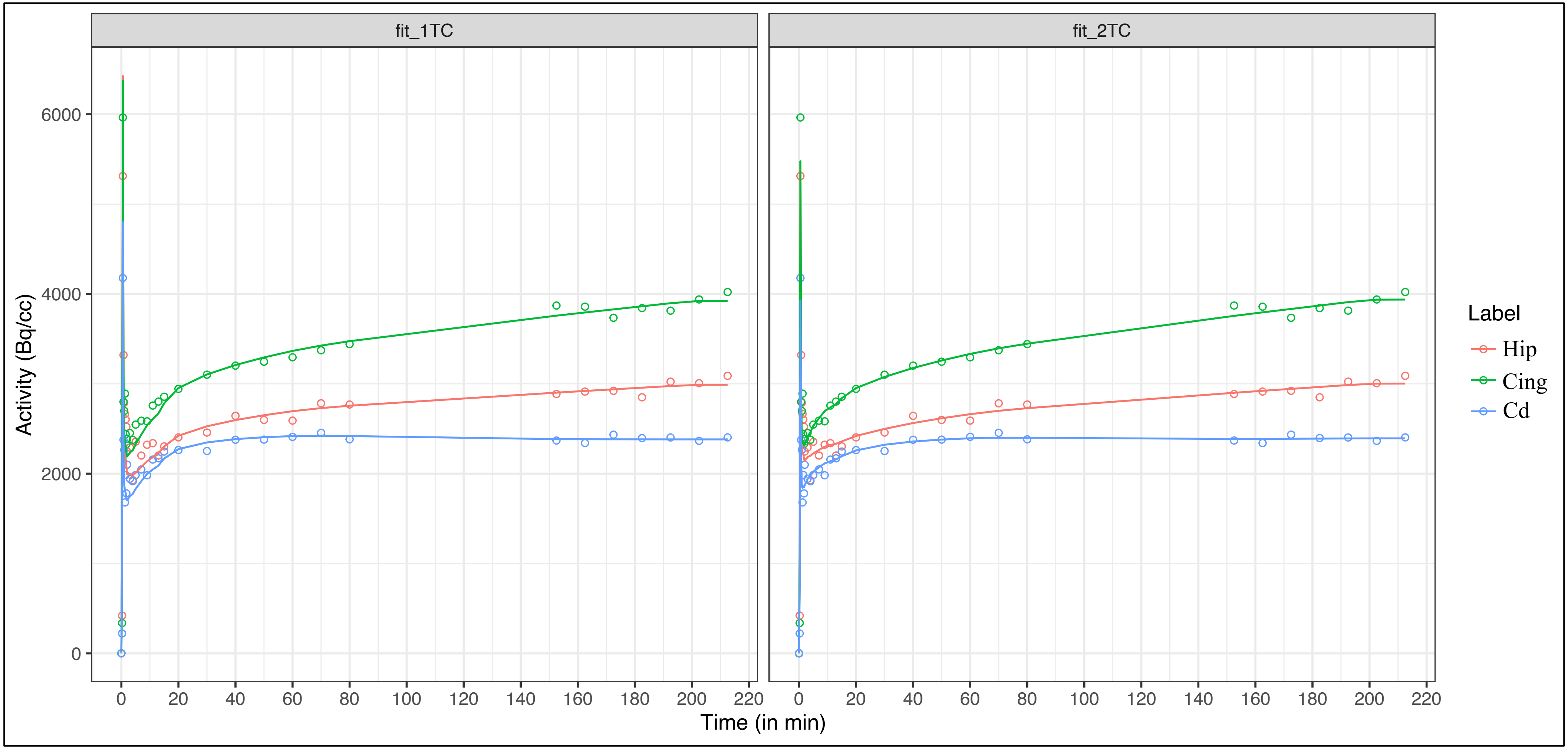

Figure S5. Measured TACs (dots) and fitted curves of one-tissue compartment (1TC) and two-tissue compartments (2TC) models for three different regions in one subject.

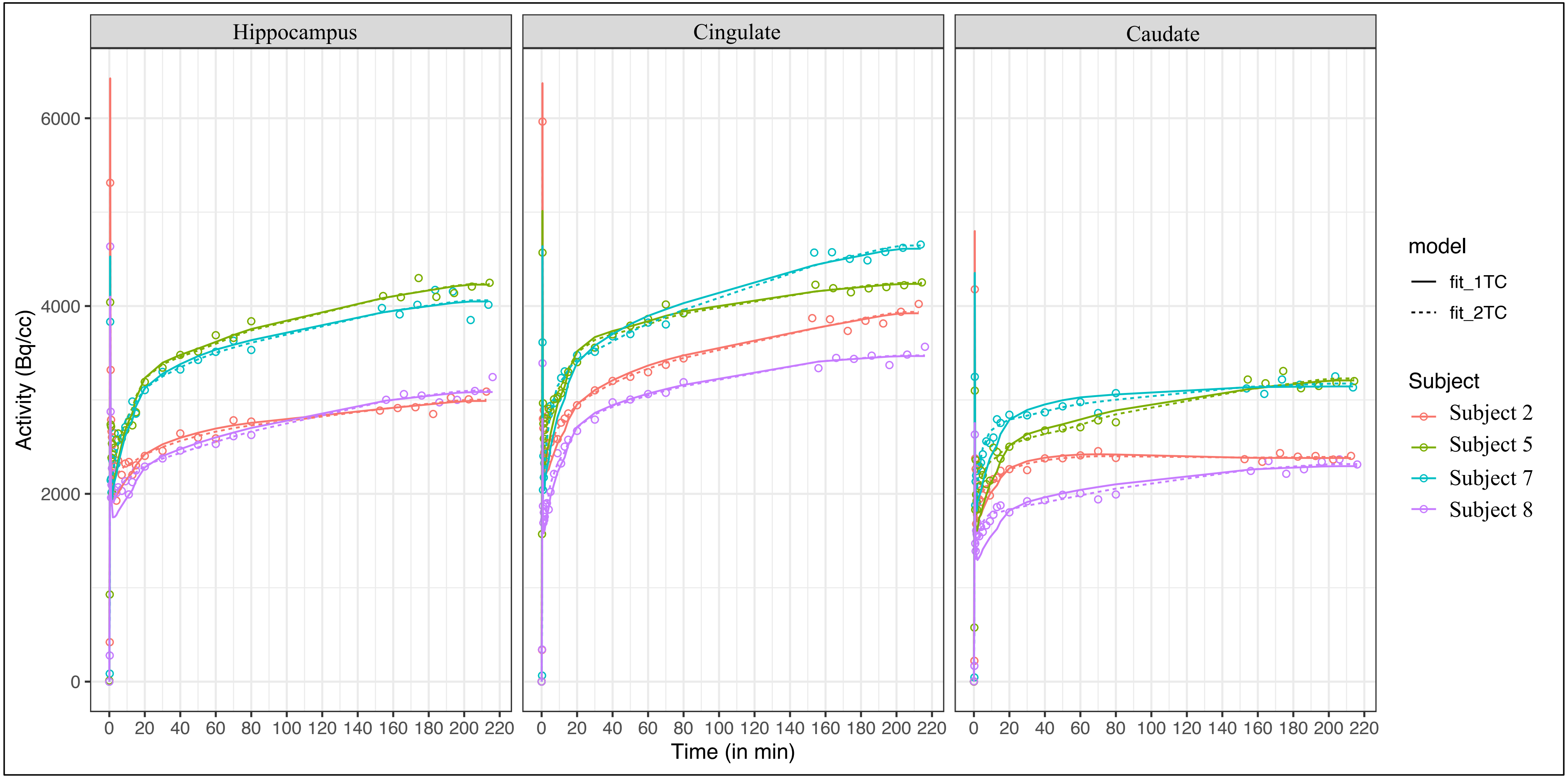

Figure S6. Measured TACs (dots) and fitted curves for of one-tissue compartment (1TC, continuous line) and two-tissue compartments (2TC, dashed line) models for three different regions for four subjects.

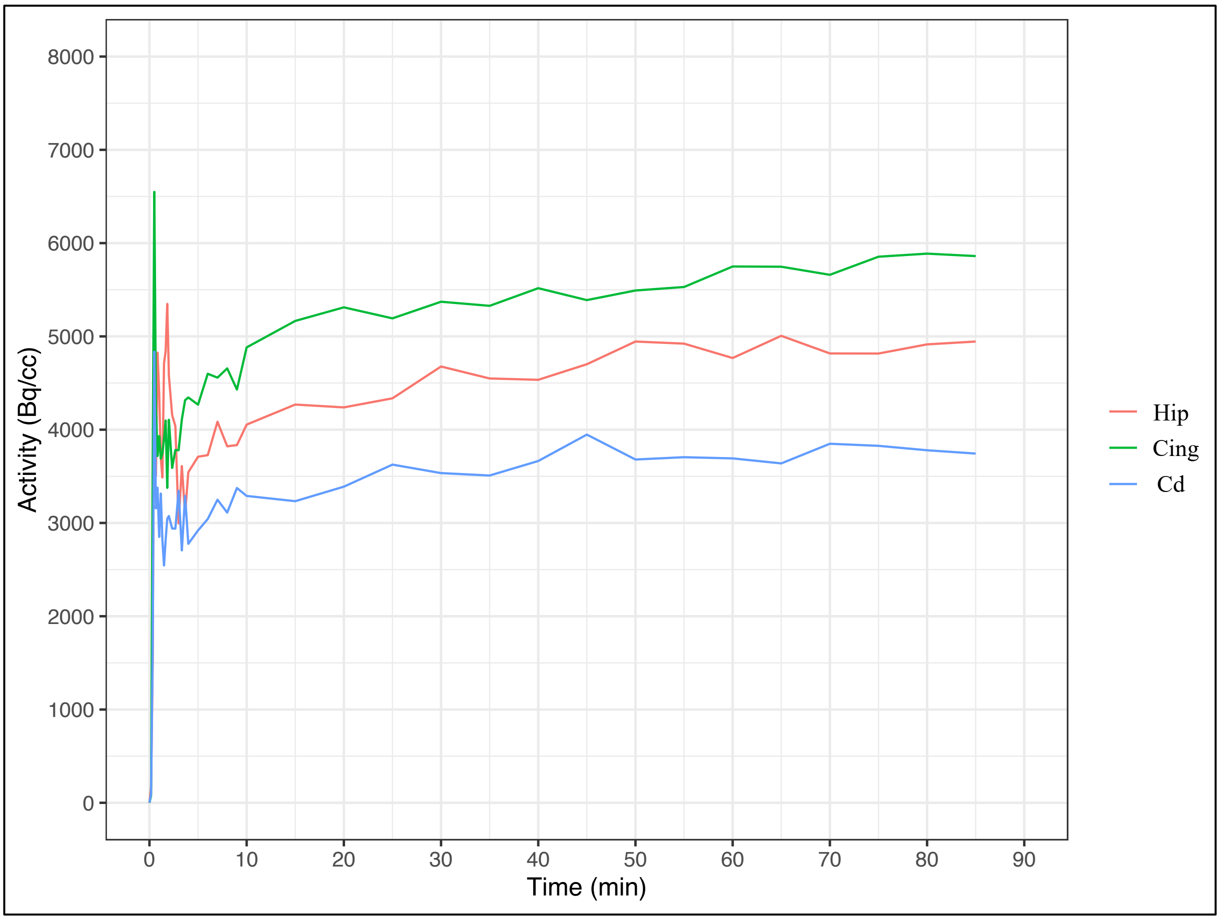

Figure S7. Example of 90 min time activity curves of three different regions (Hip: Hippocampus; Cing: Cingulate; Cd: Caudate) for one subject of the pilot PET/CT study.

Table S1. One-tissue compartment model parameters for 4 subjects assuming a time independent free fraction in plasma fp=1.76% (Vt: total volume distribution, Vb: Blood Volume). Data are presented as mean ± SD.

Regions	K1 (mL/(min*mL))	k2 (min^{-1})	Vt (mL/mL)	Vb (%)
	Cortical Region			
**Cingulate Lobe**	0.434 ± 0.084	0.004 ± 0.0005	94.3 ± 18.5	3.3 ± 0.3
**Frontal Lobe**	0.415 ± 0.078	0.005 ± 0.0003	79.5 ± 18.6	2.9 ± 0.3
**Occipital Lobe**	0.387 ± 0.078	0.006 ± 0.0002	69.9 ± 15.2	3.2 ± 0.4
**Parietal Lobe**	0.407 ± 0.085	0.005 ± 0.0002	75.6 ± 17.0	3.4 ± 0.3
**Temporal Superior Lobe**	0.410 ± 0.091	0.005 ± 0.0003	78.0 ± 18.6	3.5 ± 0.3
**Temporal Inferior Lobe**	0.374 ± 0.089	0.005 ± 0.0003	71.2 ± 17.5	2.5 ± 0.3
	Subcortical Region			
**Amygdala**	0.348 ± 0.071	0.004 ± 0.0005	90.9 ± 20.7	3.1 ± 0.6
**Central Grey Nuclei**	0.333 ± 0.081	0.005 ± 0.0005	66.2 ± 16.9	2.6 ± 0.4
**Hippocampus**	0.337 ± 0.073	0.004 ± 0.0004	84.6 ± 19.5	3.0 ± 0.5
**Insula**	0.376 ± 0.075	0.004 ± 0.0003	86.1 ± 20.9	3.2 ± 0.4
**Parahippocampal gyrus**	0.332 ± 0.061	0.005 ± 0.0004	73.0 ± 15.6	4.0 ± 0.8
**Thalamus**	0.326 ± 0.078	0.005 ± 0.0006	81.4 ± 21.8	3.4 ± 0.3
	Brainstem			
**Brainstem**	0.330 ± 0.075	0.004 ± 0.0001	82.6 ± 20.8	2.9 ± 0.4
**Dorsal Raphe Nucleus**	0.384 ± 0.083	0.004 ± 0.0006	98.3 ± 31.3	2.7 ± 0.5
**Median Raphe Nucleus**	0.360 ± 0.068	0.004 ± 0.0009	102.3 ± 38.7	2.7 ± 0.5
	Cerebellum			
**Cerebellum**	0.427 ± 0.102	0.005 ± 0.0003	78.5 ± 18.7	3.7 ± 0.4
**Cerebellum Grey Matter**	0.427 ± 0.110	0.005 ± 0.0003	78.5 ± 20.2	3.7 ± 0.4
**Cerebellum White Matter**	0.324 ± 0.081	0.005 ± 0.0003	63.9 ± 15.4	2.4 ± 0.3
**Vermis**	0.435 ± 0.112	0.006 ± 0.0006	77.9 ± 19.7	3.5 ± 0.3
	Reference region			
**Frontal Lobe White Matter**	0.259 ± 0.045	0.004 ± 0.0002	63.7 ± 14.1	1.7 ± 0.2
**Corpus Callosum**	0.183 ± 0.030	0.003 ± 0.0002	55.5 ± 12.3	1.9 ± 0.4
